# Efficacy and safety of FDA-approved IDH inhibitors in the treatment of IDH mutated acute myeloid leukemia: a systematic review and meta-analysis

**DOI:** 10.1186/s13148-023-01529-2

**Published:** 2023-07-11

**Authors:** Xiu Chen, Hongyun Xing, Xiaolu Xie, Liqiu Kou, Jun Li, Yaling Li

**Affiliations:** 1 Department of Pharmacy, The Affiliated Hospital of Southwest Medical University, Luzhou, China; 2 School of Pharmacy, Southwest Medical University, Luzhou, China; 3 Department of Hematology, The Affiliated Hospital of Southwest Medical University, Luzhou, China; 4 Department of Traditional Chinese Medicine, The Affiliated Hospital of Southwest Medical University, Luzhou, China

**Keywords:** IDH inhibitors, Ivosidenib, Enasidenib, Acute myeloid leukemia, Meta-analysis

## Abstract

**Objective:**

To systematically evaluate the efficacy and safety of FDA-approved isocitrate dehydrogenase (IDH) inhibitors in the treatment of IDH-mutated acute myeloid leukemia (AML).

**Methods:**

We used R software to conduct a meta-analysis of prospective clinical trials of IDH inhibitors in the treatment of IDH-mutated AML published in PubMed, Embase, Clinical Trials, Cochrane Library and Web of Science from inception to November 15th, 2022.

**Results:**

A total of 1109 IDH-mutated AML patients from 10 articles (11 cohorts) were included in our meta-analysis. The CR rate, ORR rate, 2-year survival (OS) rate and 2-year event-free survival (EFS) rate of newly diagnosed IDH-mutated AML (715 patients) were 47%, 65%, 45% and 29%, respectively. The CR rate, ORR rate, 2-year OS rate, median OS and median EFS of relapsed or refractory (R/R) IDH-mutated AML (394 patients) were 21%, 40%, 15%, 8.21 months and 4.73 months, respectively. Gastrointestinal adverse events were the most frequently occurring all-grade adverse events and hematologic adverse events were the most frequently occurring ≥ grade 3 adverse events.

**Conclusion:**

IDH inhibitor is a promising treatment for R/R AML patients with IDH mutations. For patients with newly diagnosed IDH-mutated AML, IDH inhibitors may not be optimal therapeutic agents due to low CR rates. The safety of IDH inhibitors is controllable, but physicians should always pay attention to and manage the differentiation syndrome adverse events caused by IDH inhibitors. The above conclusions need more large samples and high-quality RCTs in the future to verify.

**Supplementary Information:**

The online version contains supplementary material available at 10.1186/s13148-023-01529-2.

## Introduction

Acute myeloid leukemia (AML), a highly aggressive and heterogeneous hematological malignant tumor, is the most common type of acute leukemia affecting adults and has caused a large number of leukemia-related deaths [1, 2]. The median age of AML diagnosis ranged from 68 to 71 years old, indicating that most of AML occurred in the elderly [3, 4]. For patients with newly diagnosed AML, intensive induction chemotherapy consisting of cytarabine and anthracyclines (commonly known as the “7 + 3” regimen) is the standard treatment for patients suitable for intensive chemotherapy, and venetoclax combined with hypomethylated drugs is the standard treatment for patients aged > 75 years and for those not eligible for intensive chemotherapy [5, 6]. Current standard therapies may cure approximately 40–45% of young adults and 10–20% of older adults [7]. For R/R AML patients, there are currently no standard therapies and drug options are limited. The only potentially curative treatment for most R/R AML patients is allogeneic hematopoietic stem cell transplantation, which requires matched donors and may lead to graft-versus-host disease and severe fatal infections [8, 9]. Disappointingly, relapsed/refractory (R/R) AML patients have a very poor prognosis, with cure rates of no more than 10% [7]. So, the treatment of R/R AML patients has been a major challenge in the field of AML. With the development of next-generation sequencing in recent years, we have a better understanding of the genetic characteristics of AML and identified many recurrent mutant genes related to the pathogenesis of the disease. In recent years, many molecular targeted drugs based on mutant genes (IDH, FLT3) have shown good curative effect in people with specific gene mutations [10], bringing hope for R/R AML patients.

Mutations in isocitrate dehydrogenase genes (IDH1 and IDH2) occur in about 20% of AML patients [11]. This mutation can lead to the production of carcinogenic metabolite R-2-hydroxyglutaric acid (R-2-HG), which leads to DNA hypermethylation and inhibition of hematopoietic stem cell differentiation [12]. IDH inhibitors can benefit IDH-mutated AML patients by inhibiting isocitrate dehydrogenase, and show good clinical efficacy in IDH-mutated AML patients [13]. Venugopalet et al. [14] reported in a clinical trial that about 26% of R/R AML patients with IDH mutations achieved complete remission after receiving IDH inhibitors. So far, FDA has approved two IDH inhibitors to treat AML patients with IDH mutations. Enasidenib was approved in 2017 for the treatment of adult with R/R AML with susceptible IDH2 mutation [15]. Ivosidenib was approved in 2018 for the treatment of patients with R/R AML harboring IDH1 mutations and subsequently in 2019 for the treatment of newly diagnosed AML who are ≥ 75 years of age or are ineligible for intensive chemotherapy with a susceptible IDH1 mutation [16, 17].

IDH inhibitors are very promising drugs for AML patients with IDH mutations, especially for the elderly and R/R AML patients with IDH mutations. However, there are some concerns about IDH inhibitors. For example, Elihu Estey et al. [18] doubted whether it was warranted for FDA to approve ivosidenib for IDH1-mutated newly diagnosed elderly AML based on CR + CRh without survival or event-free survival data [17]. Differentiation syndrome caused by IDH inhibitors has also aroused some concerns [19]. There is an urgent need for high-quality research to evaluate the efficacy and safety of IDH inhibitors to solve these doubts. However, most of the currently published studies on IDH inhibitors in IDH-mutated AML patients are single-arm clinical studies and retrospective studies, and individual studies cannot provide strong evidence due to their single-center and small sample size limitations. In this meta-analysis, we aimed to systematically evaluate the efficacy and safety of FDA-approved IDH inhibitors in the treatment of AML patients with IDH mutations by pooling and analyzing treatment response, survival, and safety-related data from relevant published prospective clinical studies. We hope to provide more evidence for the safe and rational use of IDH inhibitors and provide some reference for subsequent related research.

## Methods

This study followed the Preferred Reporting Items for Systematic Reviews and Meta-Analyses (PRISMA) guidelines.

### Literature search

We searched Web of Science, Embase, Cochrane Library, PubMed, and ClinicalTrials.gov databases to identify relevant prospective clinical studies published from inception to November 15, 2022, without language restrictions. We searched for the following keywords: acute myeloid leukemia, AML, acute myelogenous leukemia, enasidenib, AG221, ivosidenib, AG120. In addition, we have reviewed the references of the retrieved literature to identify any possible relevant studies.

### Selection criteria

We searched for prospective clinical studies of IDH inhibitors in the treatment of AML patients with IDH mutations. Inclusion criteria were based on the PICO-framework. Population (P): AML patients with IDH mutations. Intervention (I): Treatments containing IDH inhibitors (enasidenib or ivosidenib). Comparison (C): Placebo or treatments without IDH inhibitors (enasidenib or ivosidenib). Response outcome (O): complete remission (CR) rate; overall response rate (ORR) included complete remission, complete remission with incomplete hematologic or platelet recovery, partial remission, and morphologic leukemia-free state; Survival Outcome (O): 2-year overall survival (OS) rate; 2-year event-free survival (EFS) rate, median overall survival, median event-free survival. Safety outcome (O): all grade adverse events during the whole treatment period; grade ≥ 3 adverse events during the whole treatment period.

The following are our criteria for excluding literature: (a) retrospective studies; (b) review and guideline; (c) with unavailable study data; (d) investigation; (e) conference articles; (f) repeated reported articles; (g) bridging studies; (h) maintenance therapy after allogeneic hematopoietic cell transplantation. When there is a dispute between two reviewers, the decision is made by the third reviewer (YL).

### Data extraction

Two reviewers (XC and XX) independently extracted the following information: author/year, national clinical trial (NCT) number, study design, nation, stage, IDH mutations type, median age, sample size, interventions, median follow-up, outcomes.

### Quality assessment

We assessed the quality of included RCTs and non-randomized prospective cohort studies according to the modified Jadad scale and the Methodological Index for Non-Randomized Studies Trials (MINORS) [20], respectively.

### Statistical analysis

R software version 4.2.0 was used to statistically analyze treatment response, survival, and safety results in newly diagnosed and R/R AML patients with IDH mutations treated with IDH inhibitors. We analyzed these results separately for newly diagnosed IDH-mutated AML and R/R AML with IDH mutations, and subsequently performed subgroup analyses according to medication regimen (IDH inhibitor combination therapy and IDH inhibitor monotherapy). The effect size of all pooled results were expressed by 95% confidence interval (CL), which has an upper limit and a lower limit. Both random-effects and fixed-effects models were used to draw forest plots. The Cochrane Q chi-square test and *I*^2^ statistic were used to examine the heterogeneity across studies. If *I*^2^ indicates that there is no statistical heterogeneity between the studies (*I*^2^ < 50%), we use the fixed effect model for analysis. Otherwise, we use random effect model for analysis. For the subgroup analysis results with high heterogeneity (*I*^2^ > 75%), we comprehensively analyzed the sources of heterogeneity. Publication bias was evaluated by Egger’s and Begg’s tests. *P* < 0.05 indicates statistical significance, and there may be publication bias.

## Results

### Selection of eligible studies

**Fig. 1:**
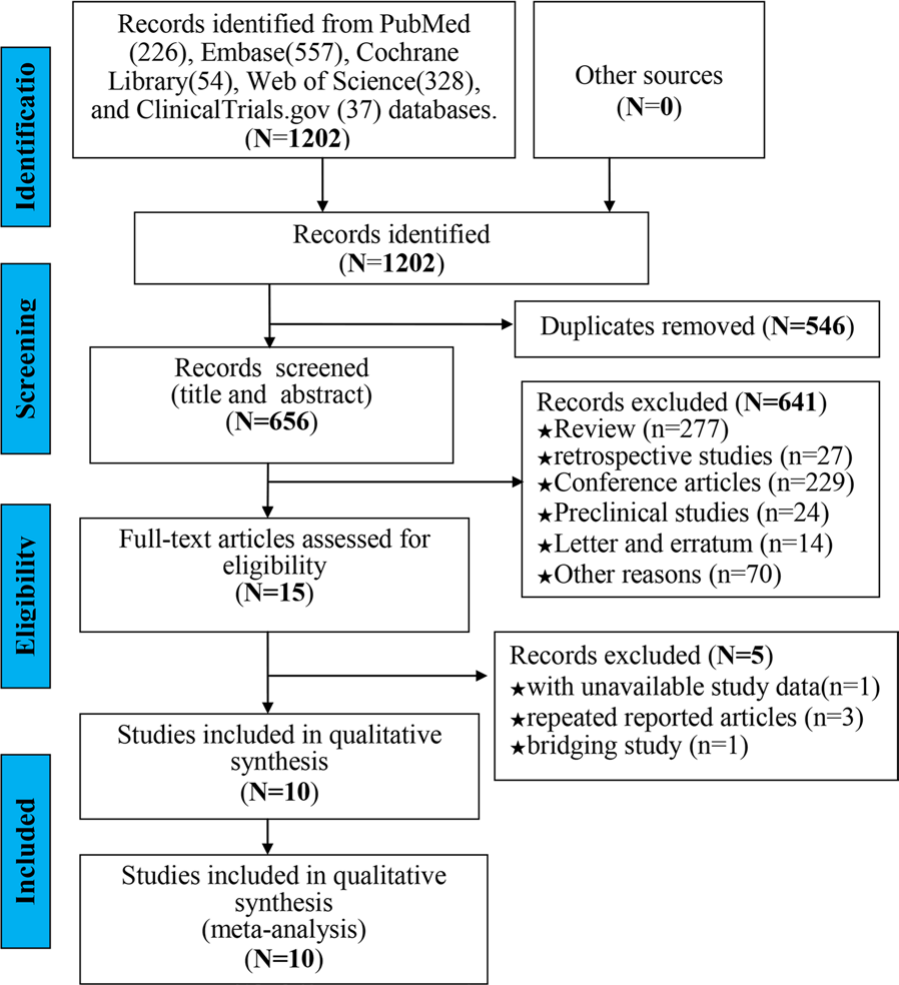
The PRISMA flowchart shows the selection process of the systematic review. The abstracts of all the studies were imported into Endnote from the indicated databases

According to the search strategy, a total of 1202 articles were identified. First, we removed 546 duplicate articles with EndNote software. Then, we excluded 641 articles after screening the title and abstract, and excluded 5 articles after reading the full text. Ultimately, 10 articles (11 cohorts) were eligible for analysis [14, 21–29]. Figure 1 shows a flow chart depicting the articles selection process.

### Characteristics of eligible studies

This meta-analysis involved 1109 IDH-mutated AML patients from 3 RCTs [21, 23, 25] and 8 prospective cohort studies (7 articles) [14, 22, 24, 26–29], including 715 R/R AML patients with IDH mutations and 394 newly diagnosed AML patients with IDH mutations. These studies were conducted in more than 10 countries (e.g. the United States, France, and Germany), and participants received 4 treatment regimens: IDH inhibitor alone (769 patients), IDH inhibitors combined with azacitidine (176 patients), IDH inhibitors combined with azacitidine and other anticancer drugs (13 patients) and IDH inhibitors combined with intensive chemotherapy (151 patients). Median age reported in the articles ranged from 62.5 to 77 years, with the majority being elderly. Table 1 shows the characteristics of randomized and non-randomized prospective clinical studies included.

**Table 1:** Characteristics of the prospective clinical studies (including randomized and non-randomized studies) and patients included in the meta-analysis

Author/year	NCT number	Study design	Nation	Stage	IDH mutations type	Median age (year)	Sample size	Interventions	Median follow-up (months)	Outcomes	Scores*
Venugopalet et al. [14]	NCT03683433	Prospective phase 2 study	United States	Newly diagnosed IDH2-mutated AML	IDH2-mutated	NA	7	Enasidenib + Azacitidine + venetoclax or FLT3i	13.1	A	15
Venugopal [14] et al	NCT03683433	Prospective phase 2 study	United States	IDH2-mutated R/R AML	IDH2-mutated	NA	19	Enasidenib + Azacitidine + venetoclax or FLT3i	NA	A	15
Montesinos et al. [21]	NCT03173248	Double-blind, randomized, placebo-controlled, phase 3 trial	United States, Australia, and Brazil et. al	Newly diagnosed IDH1-mutated AML	R132C, R132H, R132G, R132L, R132S	76.0	72	Ivosidenib + Azacitidine	12.4	A,B,C,D,E,G,H	7
DiNardo et al. [22]	NCT02677922	Prospective phase Ib study	United States, Canada, and France et. al	Newly diagnosed IDH1-mutated AML	R132C, R132H, R132L,	76.0	23	Ivosidenib + Azacitidine	16.1	A,B,H	15
DiNardo et al. [23]	NCT02677922	Open-label, randomized, phase 1b/2 trial	United States, Canada, and France et. al	Newly diagnosed IDH2-mutated AML	R140(75%), R172 (24%), data missing (1%)	75	68	Enasidenib + Azacitidine	14·9	A,B,C,E,G,H	3
Stein et al. [24]	NCT02632708	Prospective phase 1 study	United States, Germany, and Netherlands	Newly diagnosed IDH-mutated AML	IDH1-mutated and IDH2-mutated	NA	151	Enasidenib or Ivosidenib + intensive chemotherapy	NA	A,B,C	16
Botton et al. [25]	NCT02577406	Open-label, randomized, phase 3 trial	United States, Brazil, and Belgium et. al	IDH2-mutated R/R AML	IDH2-R140 (72.8%), IDH2-R172 (27.2%)	72	158	Enasidenib	NA	A,B,C,D,E,F,G,H	3
Roboz et al. [26]	NCT02074839	Prospective phase 1 study	United States and France	Newly diagnosed IDH1-mutant AML	IDH1-R132 (100%)	76.5	34	Ivosidenib	23.5	A,B,C,G,H	14
DiNardo et al. [27]	NCT02074839	Prospective phase 1 study	United States and France	IDH1-mutated R/R AML	R132C(60.8%), R132H (21.6%), R132G/L/S(15.2%), wild-type (0.8%), other (1.6%)	76.5	258	Ivosidenib	14.8	A,B,C,D,H	16
Stein et al. [28]	NCT01915498	Prospective phase 1/2 study	United States and France	IDH2-mutated R/R AML	R140(75%), R172 (25%)	68	280	Enasidenib	7.8	A,B,C,D,F,G	16
Pollyea et al. [29]	NCT01915498	Prospective phase 1/2 study	United States and France	Newly diagnosed IDH2-mutated AML	R140(67%), R172 (31%), other/missing(3%)	77	39	Enasidenib	8.4	A,B,C,D,E,F,G,H	15

A: complete remission rate B: overall response rate C: 2-year overall survival rate D: median overall survival; E: 2-year event-free survival rate; F: median event-free survival; G all grade adverse events during the whole treatment period H: grade ≥ 3 adverse events during the whole treatment period. NA: not reported; IDH: isocitrate dehydrogenase genes

*R/R* relapsed or refractory, *AML* acute myeloid leukemia, *NCT number* national clinical trial number

*Randomized studies were scored according to the modified Jadad scale, and nonrandomized study trials were scored according to methodological indices

### Evaluation of the quality of eligible studies

We assessed the quality of three [21, 23, 25] RCTs according to the modified Jadad scale (7 points in total), one [21] RCT received 7 points because of its high quality, and two other open-label RCTs received 3 points because allocation concealment and blinding were not implemented. Because no control group was set, 8 [14, 22, 24, 26–29] prospective cohort studies (7 articles) scored 0 on all three items of the MINORS scale: an adequate control group, contemporary groups, and baseline equivalence of groups. The final MINORS score (24 points in total) obtained was an average of 15.25 point, ranging between 14 and 16 points. This single-arm meta-analysis only required the arm with IDH inhibitor treatment, so blinding, allocation concealment and control groups did not affect the study results. See Additional file 1: Tables S1 and S2 for details.

### Response

#### Newly diagnosed IDH-mutated AML patients

**Fig. 2:**
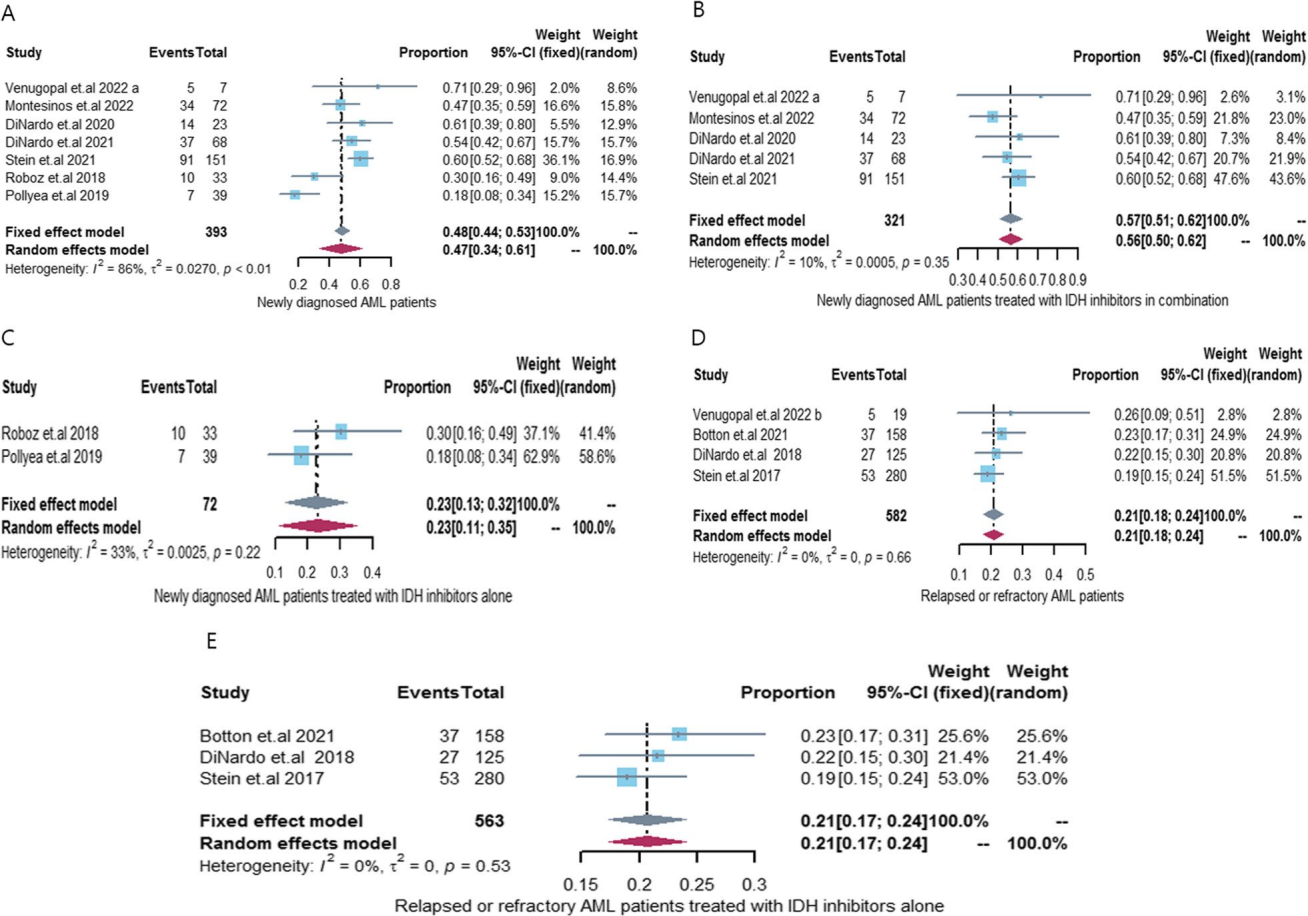
CR rate in IDH-mutant AML patients treated with IDH inhibitors. **A** Newly diagnosed AML patients treated with IDH inhibitors; **B** Newly diagnosed AML patients treated with IDH inhibitors in combination; **C** Newly diagnosed AML patients treated with IDH inhibitors alone; **D** Relapsed or refractory AML patients treated with IDH inhibitors; **E** Relapsed or refractory AML patients treated with IDH inhibitors alone. *CR* complete remission, *AML* acute myeloid leukemia, *IDH* isocitrate dehydrogenase genes, *95% CI *95% confidence interval

**Fig. 3:**
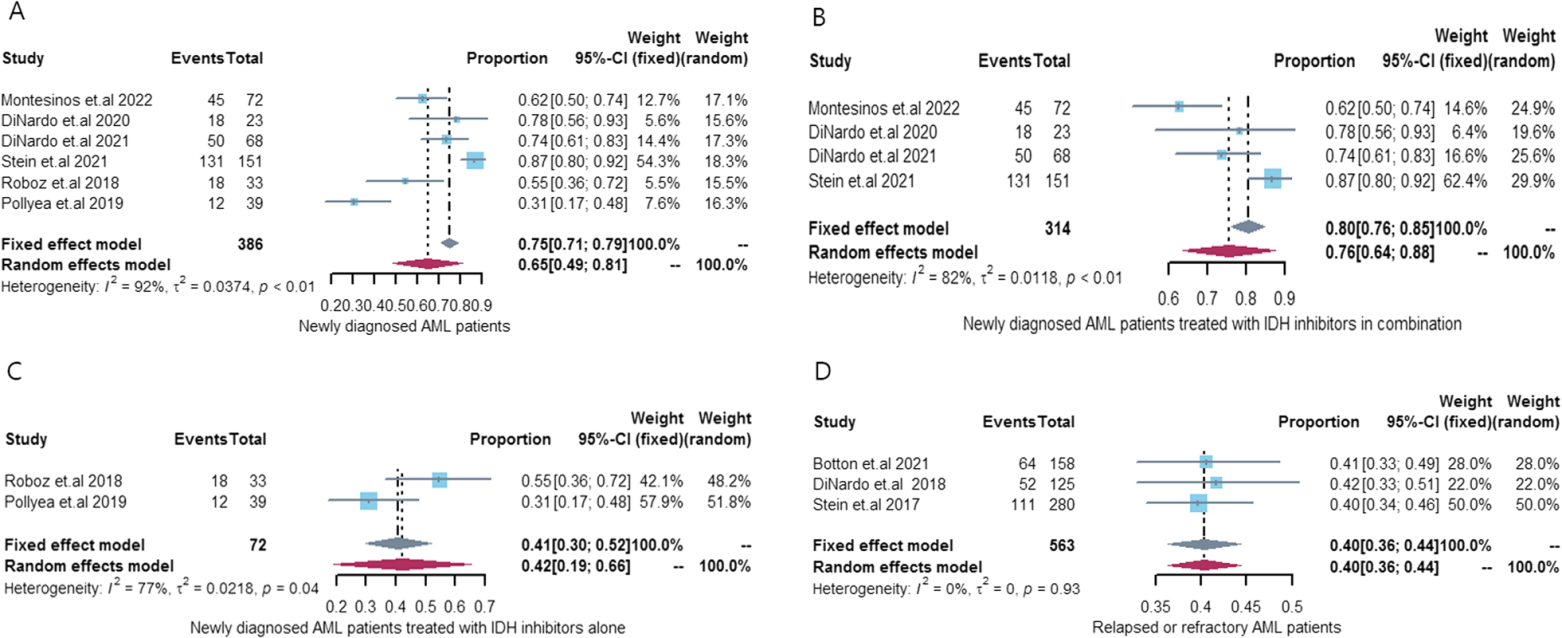
ORR rate in IDH-mutant AML patients treated with IDH inhibitors. **A** Newly diagnosed AML patients treated with IDH inhibitors; **B** Newly diagnosed AML patients treated with IDH inhibitors in combination; **C** Newly diagnosed AML patients treated with IDH inhibitors alone; **D** Relapsed or refractory AML patients treated with IDH inhibitors. *ORR* overall response, *AML* acute myeloid leukemia, *IDH* isocitrate dehydrogenase genes, *95% CI *95% confidence interval

Seven [14, 21–24, 26, 29] articles (7 cohorts) reported CR with IDH inhibitors in newly diagnosed IDH-mutated AML patients, and six [21–24, 26, 29] articles (6 cohorts) reported ORR with IDH inhibitors in newly diagnosed IDH-mutated AML patients. Our pooled CR and ORR rates were 47% [95% CI 0.34–0.61, *I*^2^ = 86%] and 65% [95% CI 0.49–0.81, *I*^2^ = 92%], respectively. Subsequent subgroup analyses showed that both CR and ORR rates were significantly higher in the IDH inhibitor combination therapy group (CR 57%, 95% CI 0.51–0.62, *I*^2^ = 10%; ORR 76%, 95% CI 0.64–0.88, *I*^2^ = 82%) than in the IDH inhibitor monotherapy group (CR 23%, 95% CI 0.13–0.32, *I*^2^ = 33%; ORR 42%, 95% CI 0.19–0.66, I^2^ = 77%). There is a high heterogeneity in ORR among newly diagnosed IDH-mutated AML patients receiving combination therapy with IDH inhibitors (*I*^2^ = 82%), which derives from the different drugs combined with IDH inhibitors (azacitidine: 3 cohorts, intensive chemotherapy: 1 cohort). And the heterogeneity was greatly reduced when a cohort of IDH inhibitors combined with intensive chemotherapy was excluded (*I*^2^ = 35%). See Additional file 1: Fig. S3 for details. Only two cohort studies have reported ORR in newly diagnosed IDH-mutated AML patients receiving IDH inhibitor monotherapy, and we hypothesize that different IDH inhibitors are a major source of heterogeneity. See Figs. 2 and 3 for details.

#### R/R IDH-mutated AML patients

Four [14, 25, 27, 28] articles (4 cohorts) reported CR with IDH inhibitors in R/R IDH-mutated AML patients, and our pooled CR rate was 21% [95% CI 0.18–0.24, *I*^2^ = 0%]. Venugopalet et al. [14] reported that 26.32% (5/19) R/R AML patients with IDH mutations achieved CR after receiving IDH inhibitor combination therapy. Excluding the study of Venugopalet et al. [14], the CR rate of R/R AML patients with IDH mutations who received IDH inhibitor alone was 21% [95% CI 0.17–0.24, *I*^2^ = 0%]. Therefore, we estimate that the CR rate of IDH inhibitor combined therapy may be slightly higher than that of IDH inhibitor monotherapy. See Fig. 2 for details.

Three [25, 27, 28] articles (3 cohorts) report ORR with IDH inhibitor monotherapy in R/R IDH-mutated AML patients, and our pooled ORR rate was 40% [95% CI 0.36–0.44, *I*^2^ = 0%]. No cohort reported ORR in R/R IDH-mutated AML patients treated with IDH inhibitor combination therapy. See Fig. 3 for details.

### Survival

#### Newly diagnosed IDH-mutated AML patients

**Fig. 4:**
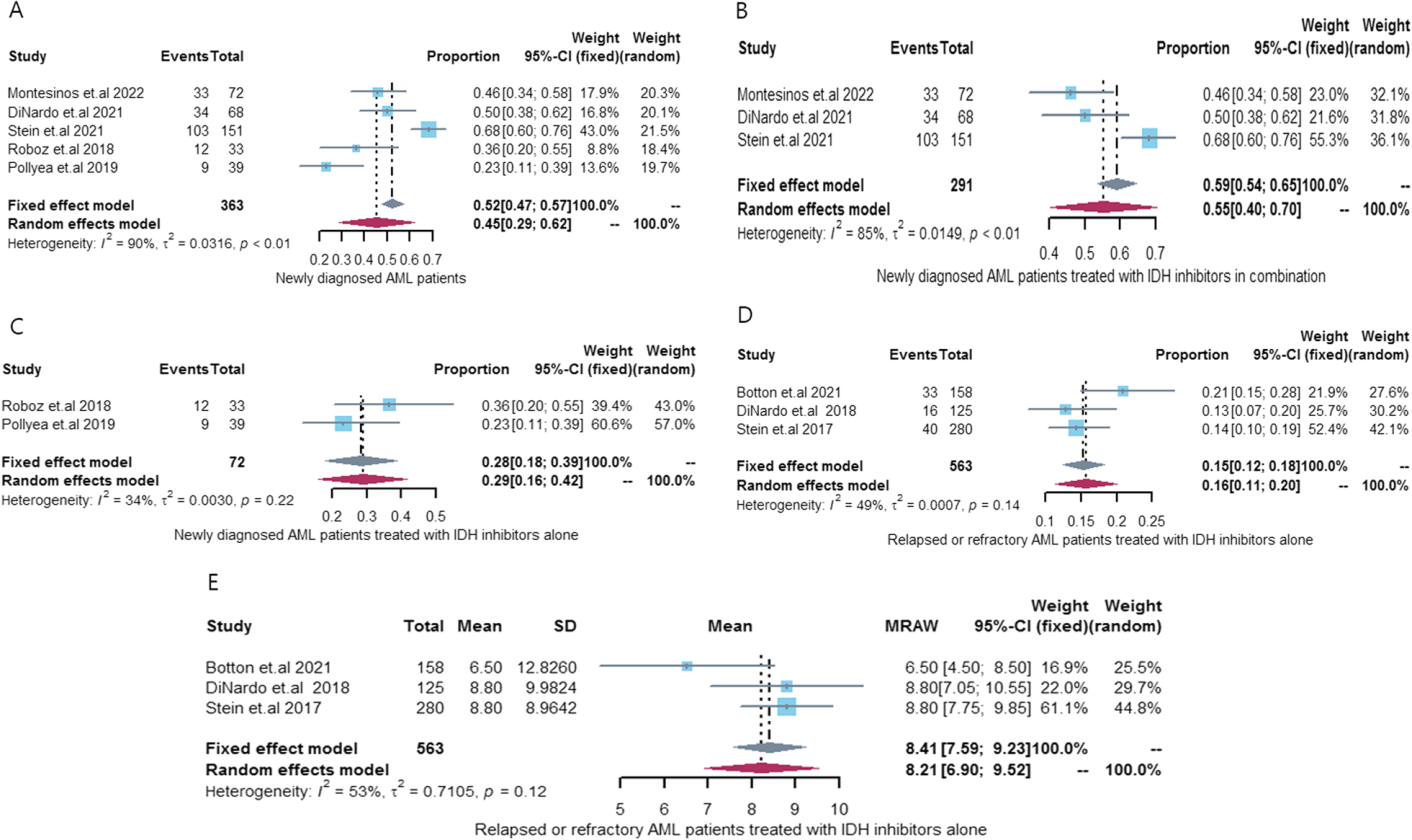
OS in IDH-mutant AML patients treated with IDH inhibitors. **A** 2-year OS rate in newly diagnosed AML patients treated with IDH inhibitors; **B** 2-year OS rate in newly diagnosed AML patients treated with IDH inhibitors in combination; **C** 2-year OS rate in newly in newly diagnosed AML patients treated with IDH inhibitors alone; **D** 2-year OS rate in relapsed or refractory AML patients treated with IDH inhibitors alone; **E** Median OS in relapsed or refractory AML patients treated with IDH inhibitors alone. *OS* overall survival, *AML* acute myeloid leukemia, *IDH* isocitrate dehydrogenase genes, *95% CI* 95% confidence interval

Five [21, 23, 24, 26, 29] articles (5 cohorts) reported the 2-year OS of IDH inhibitors in patients with newly diagnosed IDH-mutated AML, and our pooled 2-year OS rate was 45% [95% CI 0.29–0.62, *I*^2^ = 90%]. Subsequent subgroup analysis showed that the 2-year OS rate was significantly higher in the IDH inhibitor combination therapy group (OS 55%, 95% CI 0.40–0.70, *I*^2^ = 85%) than in the IDH inhibitor monotherapy group (OS 28%, 95% CI 0.18–0.39, *I*^2^ = 34%). There is a high heterogeneity in 2-year OS rate among newly diagnosed IDH-mutated AML patients receiving combination therapy with IDH inhibitors (*I*^2^ = 85%), which derives from the different drugs combined with IDH inhibitors (azacitidine: 2 cohorts, intensive chemotherapy: 1 cohort). And heterogeneity disappeared when IDH inhibitors combined with standard treatment cohort were excluded (*I*^2^ = 0%). See Additional file 1: Fig. S4 for details. One article [21] reported the median OS of IDH inhibitor combination therapy in patients with newly diagnosed IDH-mutated AML and one article [29] reported the median OS of IDH inhibitor monotherapy in patients with newly diagnosed IDH-mutated AML. Because there were too few relevant articles, we did not perform a meta-analysis of median OS. See Fig. 4 for details.

**Fig. 5:**
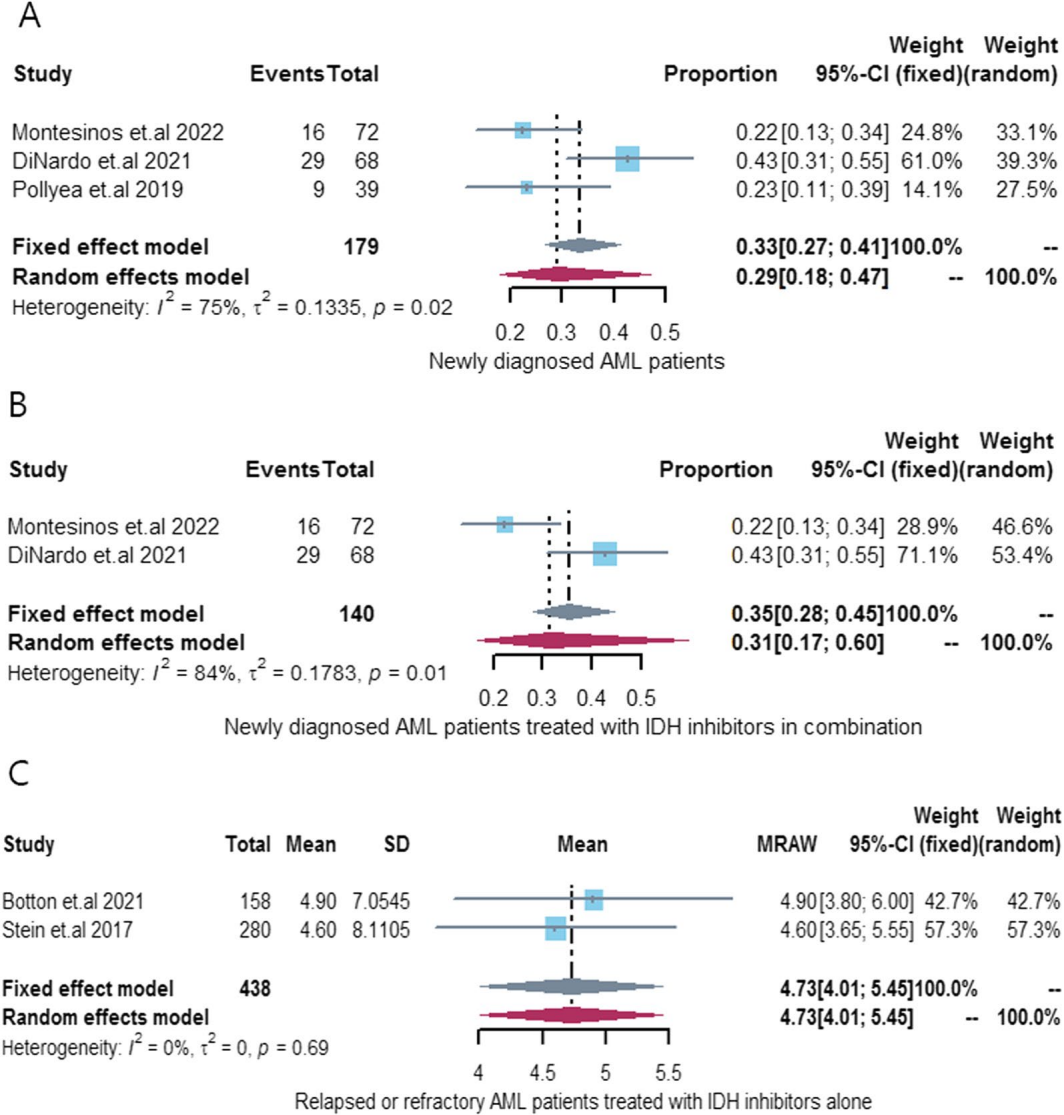
EFS in IDH-mutant AML patients treated with IDH inhibitors. **A** 2-year EFS rate in newly diagnosed AML patients treated with IDH inhibitors; **B** 2-year EFS rate in newly diagnosed AML patients treated with IDH inhibitors in combination; **C** Median EFS in relapsed or refractory AML patients treated with IDH inhibitors alone. *EFS* event-free survival, *AML* acute myeloid leukemia, *IDH* isocitrate dehydrogenase genes, *95% CI* 95% confidence interval

Three articles [21, 23, 29] (3 cohorts) reported the 2-year EFS of IDH inhibitors in patients with newly diagnosed IDH-mutated AML, and our pooled 2-year EFS rate was 29% [95% CI 0.18–0.47, *I*^2^ = 75%]. The 2-year EFS rate for IDH-inhibitor combination therapy in newly diagnosed IDH-mutated AML patients was 31% [95% CI 0.17–0.60, *I*^2^ = 84%], and the 2-year EFS rate for IDH inhibitor monotherapy in newly diagnosed IDH-mutated AML patients was not pooled due to only one relevant article. Because only Pollyea et al. [29] reported median EFS with IDH inhibitors in newly diagnosed IDH-mutated AML patients, we did not perform a meta-analysis of median EFS. See Fig. 5 for details.

#### R/R IDH-mutated AML patients

Three [25, 27, 28] articles (3 cohorts) reported the 2-year OS and median OS of IDH inhibitor monotherapy in R/R IDH-mutated AML patients. Our pooled 2-year OS rate and median OS were 15% [95% CI 0.12–0.18, *I*^2^ = 49%] and 8.21 months [95% CI 6.90–9.52, *I*^2^ = 53%], respectively. No article reported the OS results of IDH-inhibitor combination therapy in IDH-mutated AML patients. See Fig. 4 for details.

Two [25, 28] articles (2 cohorts) reported median EFS with IDH inhibitors monotherapy in R/R IDH-mutated AML, and our pooled median EFS was 4.73 months [95% CI 4.01–5.45, *I*^2^ = 0%]. Only 1 article [25] reported 2-year EFS for IDH inhibitor monotherapy in R/R IDH-mutated AML patients, so we did not perform a meta-analysis of 2-year EFS rates. No article reported the EFS results of IDH-inhibitor combination therapy in IDH-mutated AML patients. See Fig. 5 for details.

### Safe

We performed a single-arm meta-analysis of 7 [21–23, 25–27, 29] articles reporting grade ≥ 3 adverse events and 6 [21, 23, 25, 26, 28, 29] articles reporting all-grade adverse events. Common all-grade adverse events and their incidences were nausea (30%), blood bilirubin increased (24%), diarrhea (24%), constipation (23%), vomiting (21%), thrombocytopenia(20%), neutropenia(18%), anemia (17%), decreased appetite (17%), electrocardiogram QT prolongation (15%), febrile neutropenia (15%), fatigue(15%), differentiation syndrome (13%), dyspnea(11%), rash(10%), dysgeusia (9%) and leukocytosis (8%). Common grade ≥ 3 adverse events and their incidences were neutropenia (24%), thrombocytopenia (21%), anaemia (14%), febrile neutropenia (12%), sepsis (10%), blood bilirubin increased (8%), pneumonia (8%), electrocardiogram QT prolongation (6%), differentiation syndrome (5%), platelet count decreased (4%), fatigue(3%), nausea (2%), diarrhea (2%), decreased appetite (1%), vomiting (1%) and leukocytosis (1%).

### Publication bias

We performed publication bias analysis of all results using Egger’s and Begg’s tests. Eegger’s test suggests there may be publication bias in the 2-year OS rate in newly diagnosed IDH-mutated AML patients treated with IDH inhibitors (*p* = 0.0432) and therefore should be interpreted cautiously in conjunction with other results. For other results, the Egger’s test and Begg’s test did not suggest the possibility of publication bias. See Additional file 1: Table S3 for details.

## Discussion

We performed a comprehensive analysis of the efficacy of two IDH inhibitor regimens (IDH inhibitor combination and IDH inhibitor alone) in two populations (newly diagnosed IDH-mutated AML patients and R/R IDH-mutated AML patients) based on treatment response and survival. This is the first systematic review and meta-analysis to evaluate the efficacy and safety of IDH inhibitors in IDH-mutated AML patients. At present, other drug regimens for the treatment of AML patients with IDH mutations mainly include cytarabine and anthracycline induction/consolidation, and venetoclax alone or in combination [30]. We aimed to comprehensively discuss the efficacy and safety of IDH inhibitors in IDH-mutated AML patients by combining the results of our meta-analysis with other current therapeutic agents for IDH-mutated AML patients.

### Therapeutic effect of IDH inhibitor on newly diagnosed AML patients with IDH mutations

Since Mardis et al. first reported AML patients with IDH1 and IDH2 mutations in 2009 [31], researchers have been interested in the treatment of AML patients with IDH mutations. According to several studies published from 2010 to 2015 (one meta-analysis study and four clinical studies) [32–36], about 50–86% of AML patients with IDH mutations achieved CR after other treatment schemes, and the 2-year OS rate was about 35–55%. In 2018, venetoclax was approved by FDA for the treatment of AML patients, which leads to apoptosis by inhibiting B-cell leukemia/lymphoma-2 (BCL2) [37]. In an RCT [38] published in the New England Journal of Medicine, the CR rate of newly diagnosed AML patients with IDH1/IDH2 mutation was 75.4% after receiving azacitidine combined with venetoclax, which was higher than all the studies included in our meta-analysis. In another study [39], 79% of newly diagnosed AML patients with IDH mutations achieved CRc (CR plus CR with incomplete hematologic recovery) after azacitidine combined with venetoclax treatment, and the 2-year OS rate was about 52.4%. Based on the above evidence and our meta-analysis results, we think that the therapeutic response of IDH inhibitor monotherapy (CR 23%, ORR 42%, 2-year OS 28%) and combination therapy (CR 57%, ORR 76%, 2-year OS 55%, 2-year EFS 31%) in patients with newly diagnosed IDH-mutated AML is not significantly better than other therapies, but IDH inhibitor combination therapy shows comparable survival results. Because there is a lack of relevant randomized controlled studies, we can only make rough comparisons in order to gain a deeper understanding of the efficacy of IDH inhibitors. It is hoped that randomized controlled trials comparing the efficacy of IDH inhibitors and traditional therapies in newly diagnosed IDH-mutated AML patients will be reported in the future. IDH inhibitor is a promising therapy, but clinicians should try to avoid using IDH inhibitor alone, and should also consider that IDH inhibitor may cause low CR rate.

Our meta-analysis showed that IDH inhibitors combined with other agents were approximately twice as effective as IDH inhibitors alone (CR 57% vs 23%; ORR 76% vs 42%; 2-year OS rate 55% vs 28%). It has been reported that IDH1 mutations may be associated with worse prognosis in AML patients and IDH2 mutations may be associated with better prognosis in AML patients [40, 41]. In the analyses of CR and ORR rates, the proportion of patients with IDH2 mutations was greater in the monotherapy regimen (CR rate: 54.17%, ORR rate: 54.17%) than in the combination regimen (CR rate: 51.71%, ORR rate: 50.64%). In the analysis of the 2-year OS rate, the proportion of patients with IDH2 mutations in the monotherapy regimen (54.17%) was similar to the proportion of patients with IDH2 mutations in the combination regimen (54.64%). See Additional file 1: Table S4 for details. Therefore, the finding that the combination regimen was much more effective than the monotherapy regimen was not caused by differences in IDH gene mutation types between the two regimens.

The studies included in our manuscript involved two combination strategies: IDH inhibitors combined with azacitidine with or without other anticancer agents, and IDH inhibitors combined with intensive chemotherapy. Impaired cellular differentiation and leukemic development in IDH-mutated AML patients are associated with 2-hydroxyglutarate (2-HG) production and DNA hypermethylation caused by IDH gene mutations. In addition, AML patients with IDH mutations may have concomitant mutations in DNMT3A [42]. Therefore, the combination of IDH inhibitors and DNA methyltransferase (DNMT) inhibitors (e.g. azacitidine) that can reduce DNA methylation is a promising regimen for the treatment of IDH-mutated AML patients. IDH inhibitors combined with intensive chemotherapy regime is the main heterogeneous source of IDH inhibitor combined therapy subgroup (ORR, 2-year OS rate), which has shown the best therapeutic effect in all the studies on IDH inhibitor combined with other drugs in the treatment of newly diagnosed IDH-mutated AML patients. IDH inhibitor combined with intensive chemotherapy may be a better treatment regime than IDH inhibitor combined with azacytidine, but there is a lack of relevant RCTs to provide evidence.

### Therapeutic effect of IDH inhibitor on R/R AML patients with IDH mutations

R/R AML Patients have been a major challenge in AML treatment. Patients with refractory or relapsed AML (R/R AML) have a very poor prognosis with a median survival of less than 6 months and a 3-year overall survival (OS) of no more than 10%. In the subgroup analysis of three clinical studies [43–45], the CR rates of venetoclax-based drug regimen in R/R AML patients with IDH mutations were 1/11, 2/12 and 3/5, respectively, but the sample size of these studies was very small. Our meta-analysis showed that IDH inhibitor monotherapy has a good prognosis in R/R AML patients with IDH mutations and could be another option for R/R AML patients with IDH mutations (CR 21%, ORR 40%, median OS: 8.21 months, median EFS: 8.21 months, 2-year OS rate: 15%). Only one [14] of our included articles reported the prognosis of R/R IDH-mutated AML patients treated with IDH inhibitors combined with other anticancer drugs, and 5 of 19 patients (26%) achieved complete remission. IDH inhibitors combined with other anticancer agents may be more effective in IDH-mutated AML than IDH inhibitors alone, but this requires better evaluation after completion of relevant ongoing and unstarted studies (e.g. NCT04774393, NCT04250051, NCT05441514).

### Safety of IDH inhibitor in the treatment of AML patients with IDH mutant

Most AML patients are elderly, and adverse events associated with anticancer drugs are important because they generally tolerate anticancer drugs poorly due to poor physical fitness. Our detailed meta-analysis of adverse events associated with IDH inhibitors showed that all grades of adverse events commonly (> 15%) occur in IDH-mutated AML patients treated with IDH inhibitors are nausea (30%), blood bilirubin increased (24%), diarrhea (24%), constipation (23%), vomiting (21%), thrombocytopenia(20%), neutropenia(18%), anemia (17%) and decreased appetite (17%), most of which are gastrointestinal adverse events. Common (> 5%) grade ≥ 3 adverse events were neutropenia (24%), thrombocytopenia (21%), anaemia (14%), febrile neutropenia (12%), sepsis (10%), blood bilirubin increased (8%), pneumonia (8%) and electrocardiogram QT prolongation (6%), most of which are haematological adverse events. Differentiation syndrome is a potentially fatal worrisome adverse event that manifests clinically as dyspnea, fever, weight gain, unexplained hypotension, acute renal failure, and pulmonary infiltrates or pleural pericardial effusion on chest radiography [46]. Approximately 13% of patients experienced all grades of differentiation syndrome and 5% experienced grade 3 or higher differentiation syndrome. For IDH differentiation syndrome, corticosteroids, symptomatic treatment and drug withdrawal can be performed according to the patient's condition, but drug withdrawal may not take effect immediately because of the long half-life of IDH inhibitors(e.g. ivosidenib, 93 h; enasidenib, 137 h) [47]. Although differentiation syndrome may be life-threatening, patients can avoid risks and gain the greatest benefit as long as clinicians pay attention to patients at any time and do a good job in the early identification and treatment of IDH differentiation syndrome [48].

### Limitations of this meta-analysis

This meta-analysis has five limitations. First of all, our meta-analysis of survival outcomes in newly diagnosed AML patients with IDH mutations and R/R AML patients with IDH mutations lacked long-term survival (e.g. 5-year, 10-year) analyses. Future clinical studies are expected to provide long-term survival outcomes. Second, differences in follow-up time and age between studies may have some impact on the results. Third, The sample size of some articles included was small. Forth, too few R/R AML patients with IDH1 mutations (0% or 22.20%) were included for efficacy analysis of IDH inhibitors in IDH-mutated R/R AML. See Additional file 1: Table S4 for details. It is expected that more relevant studies involving R/R AML patients with IDH1 mutations will be published in the future. Finally, due to the lack of relevant clinical trials, we did not perform exploratory analyses of prognostic biomarkers (e.g. epigenetic profiles of methylation and concentrations of 2-HG in serum) associated with IDH inhibitors in IDH-mutated AML patients [49, 50]. This is an important research direction of IDH inhibitors in the treatment of AML, and it is hoped that more relevant studies will be published in the future.

## Conclusion

IDH inhibitor is a promising therapy for R/R AML patients with IDH mutations, which is of great significance for individualized and precise treatment of R/R AML patients with IDH mutations. IDH inhibitors alone and in combination with other drugs have not been significantly more effective than other current therapies (e.g. venetoclax-based drug regimen) in the treatment of newly diagnosed AML patients with IDH mutations. For newly diagnosed AML patients with IDH mutations, clinicians should avoid using IDH inhibitors alone and pay attention to the low CR rate that IDH inhibitors may cause. When IDH inhibitors are used, clinicians should pay high attention to the occurrence of differentiation syndrome, and make early diagnosis and timely treatment. However, we need more large samples and high quality RCTs in the future to prove and supplement our conclusions.

## Supplementary Information

**Additional file 1**. **Figure S1**: Forest plot of all grades adverse events related to IDH inhibitors. **Figure S2**: Forest plot of grade ≥ 3 adverse events related to IDH inhibitors. **Figure S3**: The ORR rate of newly diagnosed AML patients with IDH mutation treated by IDH inhibitor combined therapy(excluding IDH inhibitors combined with intensive chemotherapy). **Figure S4**: The 2-year OS rate of newly diagnosed AML patients with IDH mutation treated by IDH inhibitor combined therapy(excluding IDH inhibitors combined with intensive chemotherapy). **Table S1**: Quality evaluation of RCTs according to modified Jadad scale. **Table S2**: Quality evaluation of non-randomized prospective cohort studies according to Methodological Index for Non-Randomized Studies Trials. **Table S3**: Egger’s and Begg's tests of all results. **Table S4**: IDH gene mutation in AML patients involved in efficacy analysis.

## Data Availability

The datasets used and/or analyzed during the current study are available from the corresponding author on reasonable request.
