# Supplementary material for: Efficacy and safety of FDA-approved IDH inhibitors in the treatment of IDH mutated acute myeloid leukemia: a systematic review and meta-analysis
Source: Clin Epigenetics. 2023 Jul 11;15:113. doi: 10.1186/s13148-023-01529-2 (PMC10334617; doi:10.1186/s13148-023-01529-2)
Supplement: Supplementary file 1 — Additional file 1. Figure S1: Forest plot of all grades adverse events related to IDH inhibitors. Figure S2: Forest plot of grade ≥ 3 adverse events related to IDH inhibitors. Figure S3: The ORR rate of newly diagnosed AML patients with IDH mutation treated by IDH inhibitor combined therapy(excluding IDH inhibitors combined with intensive chemotherapy). Figure S4: The 2-year OS rate of newly diagnosed AML patients with IDH mutation treated by IDH inhibitor combined therapy(excluding IDH inhibitors combined with intensive chemotherapy). Table S1: Quality evaluation of RCTs according to modified Jadad scale. Table S2: Quality evaluation of non-randomized prospective cohort studies according to Methodological Index for Non-Randomized Studies Trials. Table S3: Egger’s and Begg's tests of all results. Table S4: IDH gene mutation in AML patients involved in efficacy analysis. [file 13148_2023_1529_MOESM1_ESM.docx]

**Additional file 1**

**1. Figure S1.** Forest plot of all grades adverse events related to IDH inhibitors.

**2. Figure S2.** Forest plot of grade ≥ 3 adverse events related to IDH inhibitors.

**3. Figure S3.** The ORR rate of newly diagnosed AML patients with IDH mutation treated by IDH inhibitor combined therapy(excluding IDH inhibitors combined with intensive chemotherapy).

**4. Figure S4.** The 2-year OS rate of newly diagnosed AML patients with IDH mutation treated by IDH inhibitor combined therapy(excluding IDH inhibitors combined with intensive chemotherapy).

**5. Table S1.** Quality evaluation of RCTs according to modified Jadad scale.

**6. Table S2.** Quality evaluation of non-randomized prospective cohort studies according to Methodological Index for Non-Randomized Studies Trials.

**7. Table S3.** Egger’s and Begg's tests of all results.

**8. Table S4.** IDH gene mutation in AML patients involved in efficacy analysis.

**1. Figure S1.** Forest plot of all grades adverse events related to IDH inhibitors.


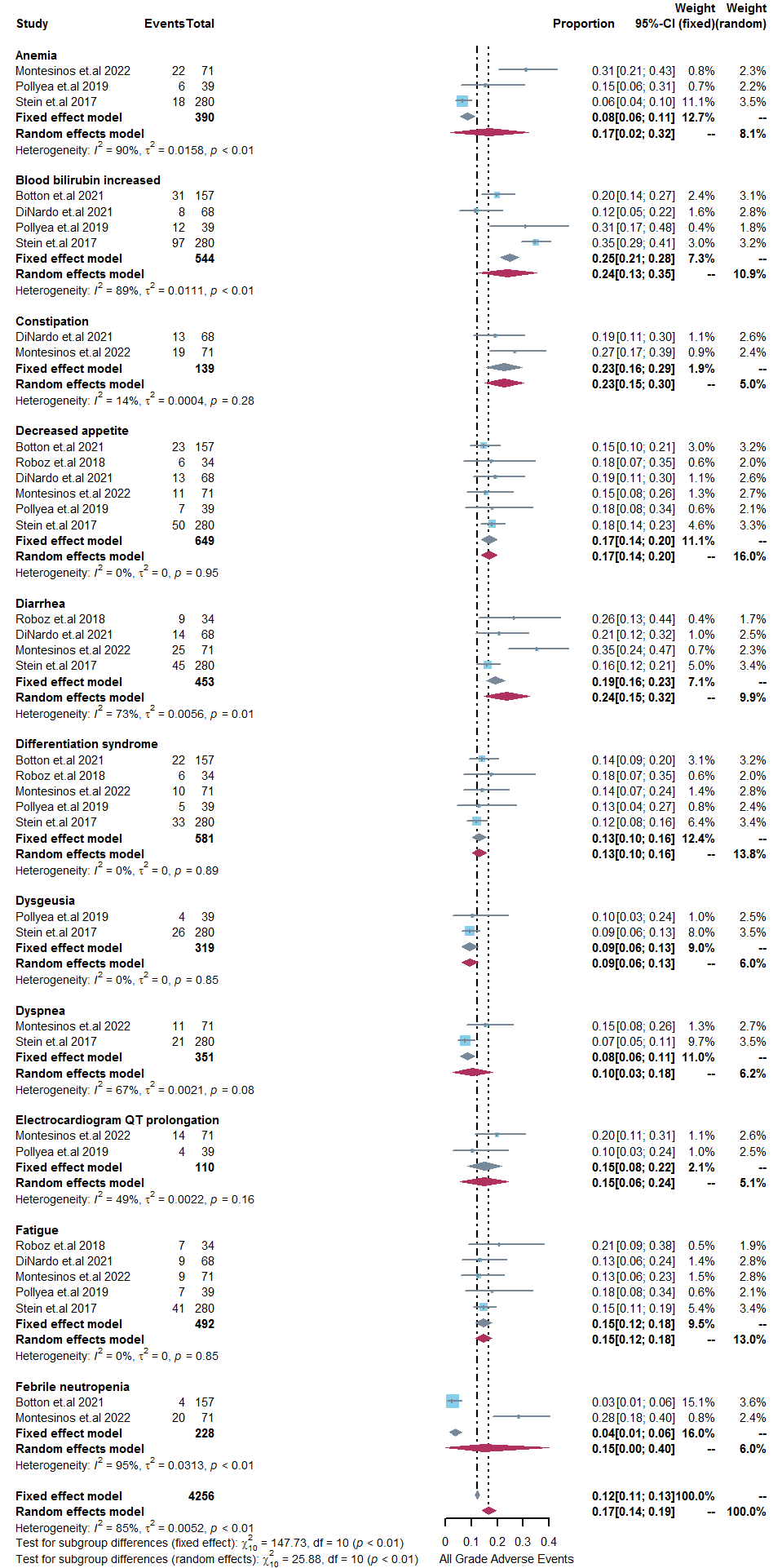


**Figure S1 A:** Forest plot of all grades adverse events related to IDH inhibitors. IDH: isocitrate dehydrogenase genes; 95% CI: 95% confidence interval.


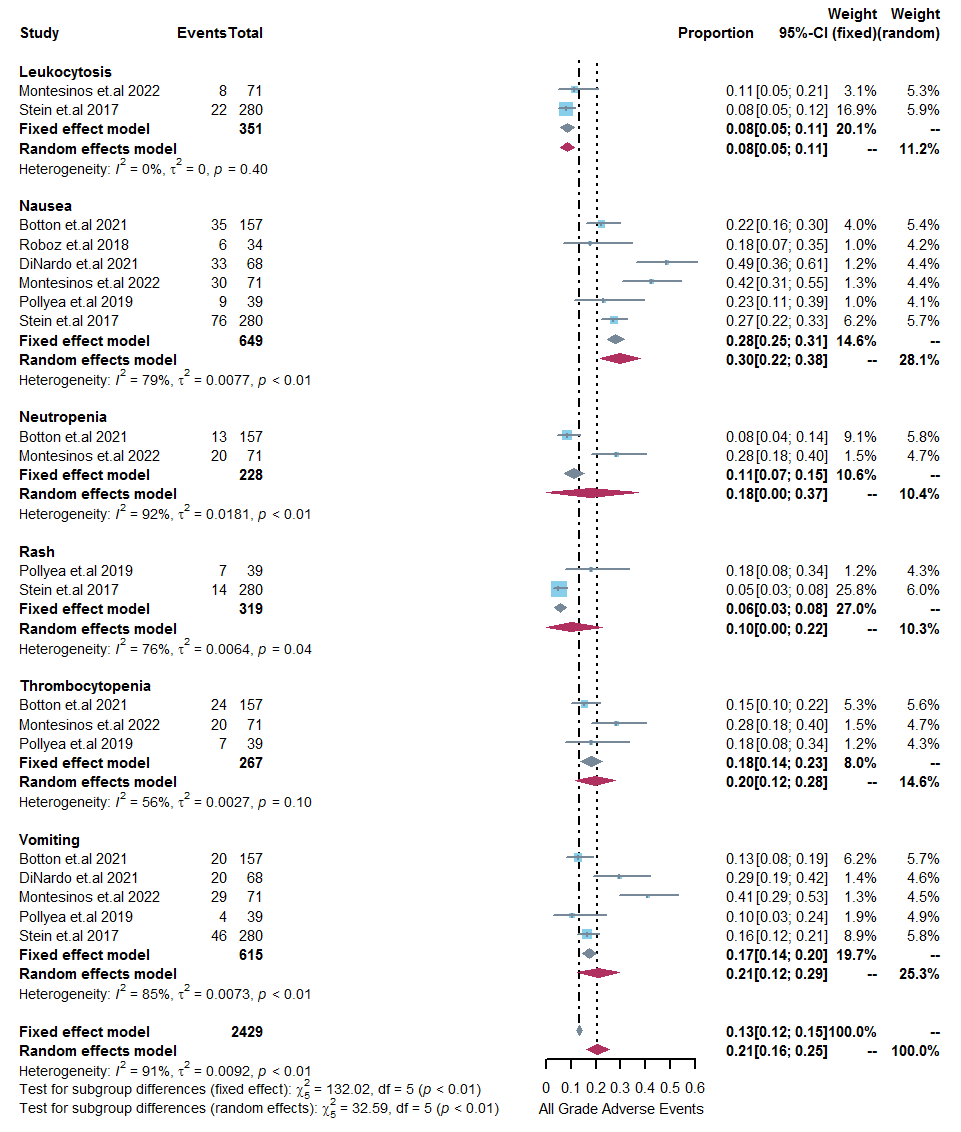


**Figure S1 B:** Forest plot of all grades adverse events related to IDH inhibitors. IDH: isocitrate dehydrogenase genes; 95% CI: 95% confidence interval.

**2. Figure S2. Forest plot of grade ≥ 3 adverse events related to IDH inhibitors.**


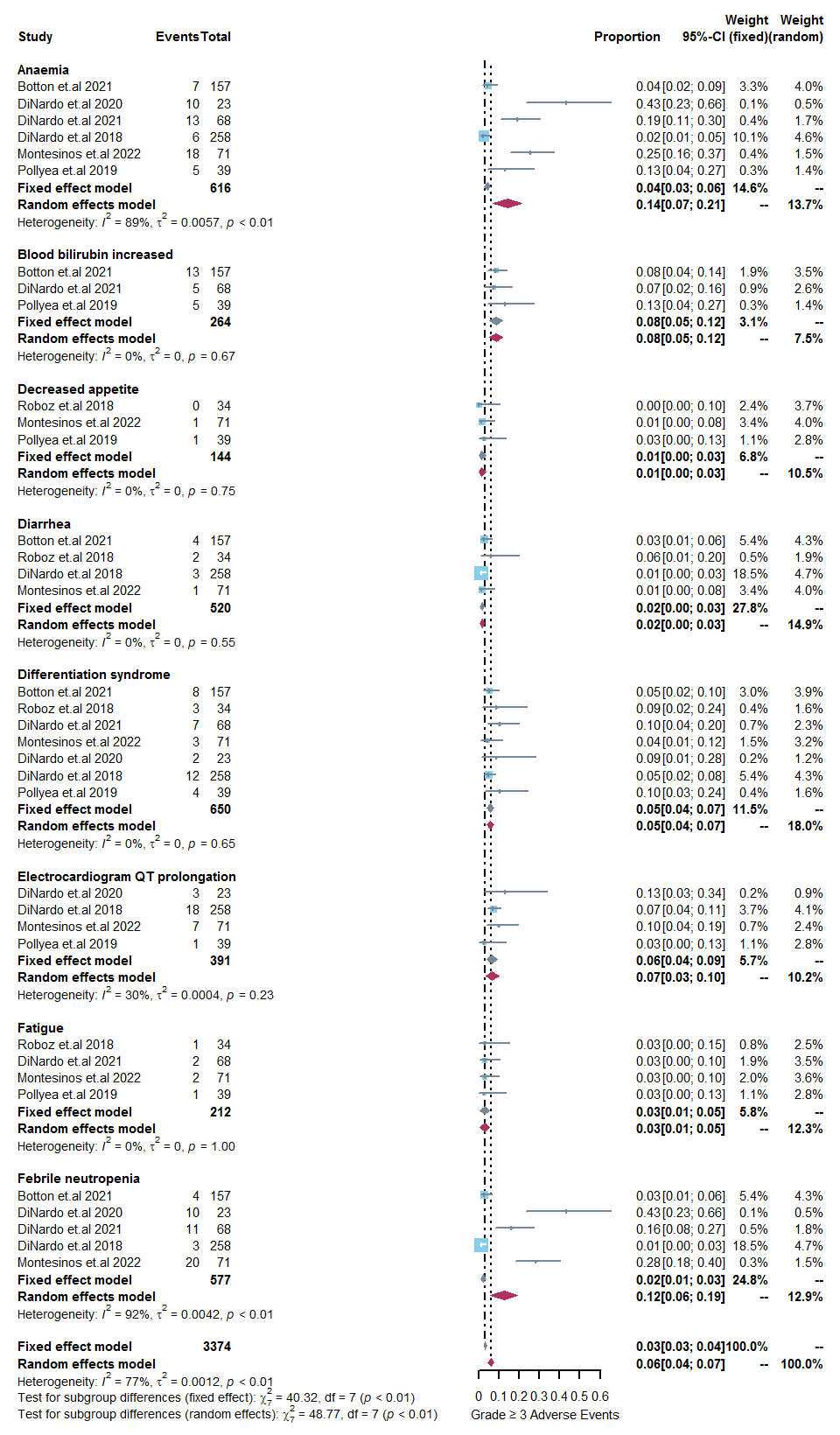


**Figure S2 A:** Forest plot of grade ≥ 3 adverse events related to IDH inhibitors. IDH: isocitrate dehydrogenase genes; 95% CI: 95% confidence interval.


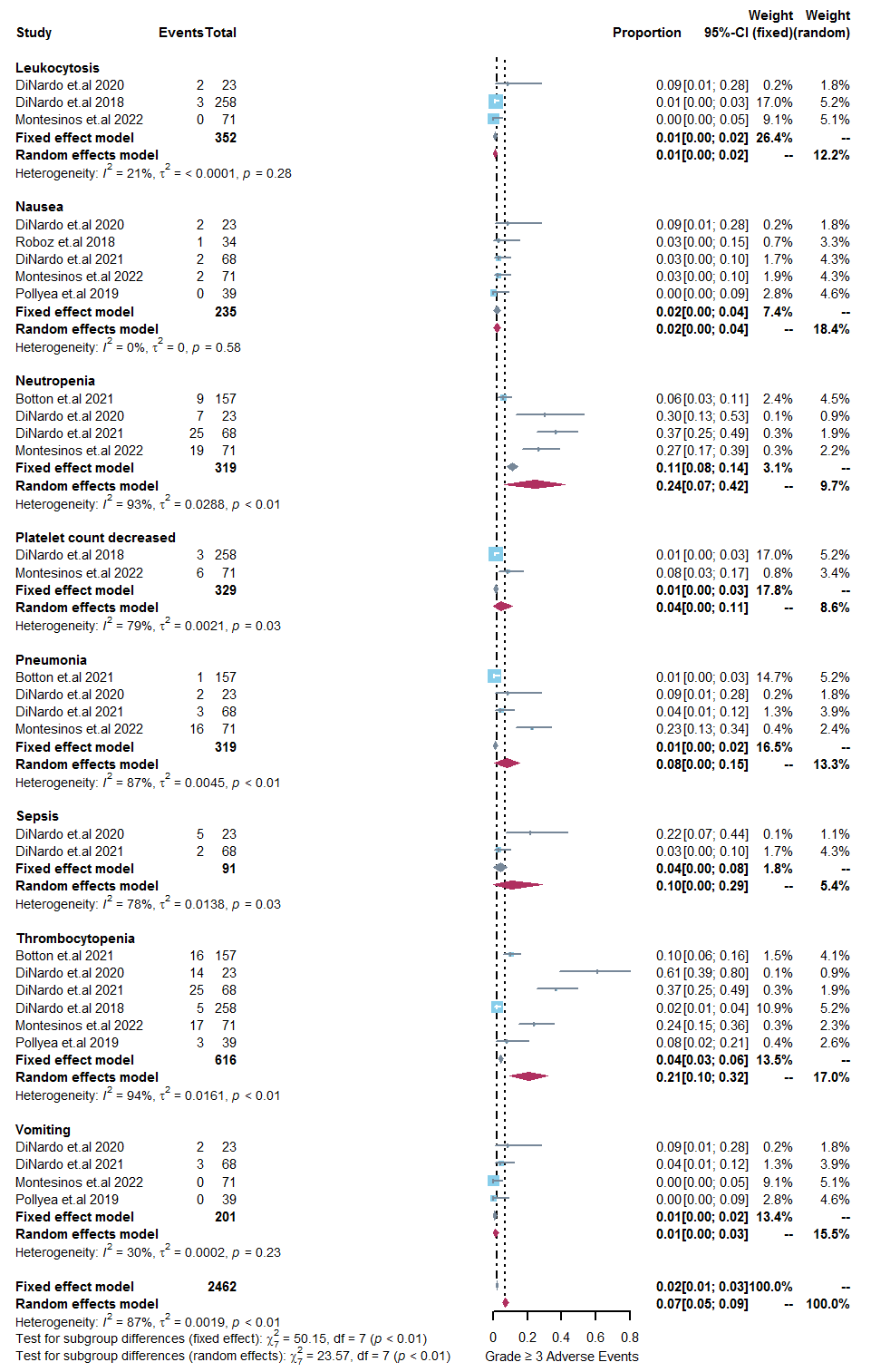


**Figure S2 B:** Forest plot of grade ≥ 3 adverse events related to IDH inhibitors. IDH: isocitrate dehydrogenase genes; 95% CI: 95% confidence interval.

**3. Figure S3.** The ORR rate of newly diagnosed AML patients with IDH mutation treated by IDH inhibitor combined therapy(excluding IDH inhibitors combined with standard therapy).

**
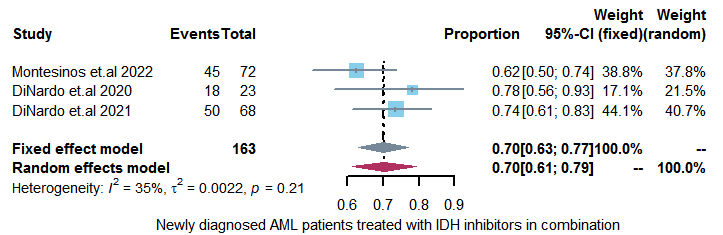
**

AML: acute myeloid leukemia; IDH: isocitrate dehydrogenase genes; 95% CI: 95% confidence interval.

**4. Figure S4.** The 2-year OS rate of newly diagnosed AML patients with IDH mutation treated by IDH inhibitor combined therapy(excluding IDH inhibitors combined with intensive chemotherapy).

**
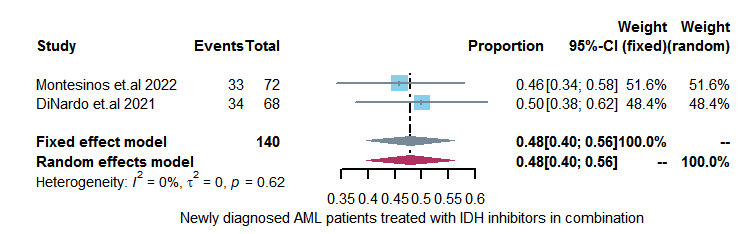
**

AML: acute myeloid leukemia; IDH: isocitrate dehydrogenase genes; 95% CI: 95% confidence interval.

**5. Table S1.** Quality evaluation of RCTs according to modified Jadad scale.

| Author/year | random sequence generation  (2 point) | randomization concealment  (2 point) | Blinding  (2 point) | Withdrawal  (1 point) | Totle  (7 point) |
| --- | --- | --- | --- | --- | --- |
| Montesinos et.al 2022 | 2 | 2 | 2 | 1 | 7 |
| DiNardo et.al 2021 | 2 | 0 | 0 | 1 | 3 |
| Botton et.al  2021 | 2 | 0 | 0 | 1 | 3 |

**6. Table S2.** Quality evaluation of non-randomized prospective cohort studies according to Methodological Index for Non-Randomized Studies Trials.

| Quality assessment for nonrandomized trials | Venugopal et.al 2022 | DiNardo  et.al 2020 | Stein  et.al2021 | Roboz et.al 2018 | DiNardo  et.al 2018 | Stein  et.al 2017 | Pollyea et.al 2019 |
| --- | --- | --- | --- | --- | --- | --- | --- |
| A clearly stated aim | 2 | 2 | 2 | 2 | 2 | 2 | 2 |
| Inclusion of consecutive patients | 2 | 2 | 2 | 2 | 2 | 2 | 2 |
| Prospective data collection | 2 | 2 | 2 | 2 | 2 | 2 | 2 |
| Endpoints appropriate to the aim of the study | 2 | 2 | 2 | 2 | 2 | 2 | 2 |
| Unbiased assessment of the study endpoint | 0 | 0 | 0 | 0 | 0 | 0 | 0 |
| A follow-up period appropriate to the aims of study | 2 | 2 | 2 | 2 | 2 | 2 | 2 |
| Less than 5% loss to follow-up | 2 | 2 | **2** | 1 | 2 | 2 | 2 |
| Prospective calculation of the sample size | 1 | 1 | 2 | 1 | 2 | 2 | 1 |
| An adequate control group | 0 | 0 | 0 | 0 | 0 | 0 | 0 |
| Contemporary groups | 0 | 0 | 0 | 0 | 0 | 0 | 0 |
| Baseline equivalence of groups | 0 | 0 | 0 | 0 | 0 | 0 | 0 |
| Adequate statistical analyses | 2 | 2 | 2 | 2 | 2 | 2 | 2 |
| Total score | 15 | 15 | 16 | 14 | 16 | 16 | 15 |

**7. Table S3.** Egger’s and Begg's tests of all results.

|  | Egger | Begg |
| --- | --- | --- |
| **CR rate in IDH-mutant AML patients treated with IDH inhibitors** | | |
| Newly diagnosed AML patients treated with IDH inhibitors | 0.7828 | 0.8806 |
| Newly diagnosed AML patients treated with IDH inhibitors in combination | 0.8046 | 0.6242 |
| Newly diagnosed AML patients treated with IDH inhibitors alone | NA | NA |
| Relapsed or refractory AML patients treated with IDH inhibitors | 0.2584 | 0.4969 |
| Relapsed or refractory AML patients treated with IDH inhibitors alone. | 0.3233 | 0.6015 |
| **ORR rate in IDH-mutant AML patients treated with IDH inhibitors** | | |
| Newly diagnosed AML patients treated with IDH inhibitors | 0.0687 | 0.1885 |
| Newly diagnosed AML patients treated with IDH inhibitors in combination | 0.2607 | 0.4969 |
| Newly diagnosed AML patients treated with IDH inhibitors alone | NA | NA |
| Relapsed or refractory AML patients treated with IDH inhibitors alone. | 0.1486 | 0.1172 |
| **OS in IDH-mutant AML patients treated with IDH inhibitors** | | |
| 2-year OS rate in newly diagnosed AML patients treated with IDH inhibitors. | 0.0432 | 0.1416 |
| 2-year OS rate in newly diagnosed AML patients treated with IDH inhibitors in combination | 0.1245 | 0,6015 |
| 2-year OS rate in newly diagnosed AML patients treated with IDH inhibitors alone | NA | NA |
| 2-year OS rate in relapsed or refractory AML patients treated with IDH inhibitors alone | 0.6510 | 0.6015 |
| Median OS in relapsed or refractory AML patients treated with IDH inhibitors alone | 0.4948 | 0.1172 |
| **EFS in IDH-mutant AML patients treated with IDH inhibitors** | | |
| 2-year EFS rate in newly diagnosed AML patients treated with IDH inhibitors. | 0.2750 | 0.6015 |
| 2-year EFS rate in newly diagnosed AML patients treated with IDH inhibitors in combination | NA | NA |
| Median EFS in relapsed or refractory AML patients treated with IDH inhibitors alone | NA | NA |

IDH: isocitrate dehydrogenase; CR: complete remission; ORR: overall response, including complete remission, complete remission with incomplete hematologic or platelet recovery, partial remission, and morphologic leukemia-free state; OS: overall survival; EFS: event-free survival; NA: The number of studies is too small to get results; AML: acute myeloid leukemia. *P* < 0.05 indicates a possible publication bias.

**8. Table S4.** IDH gene mutation in AML patients involved in efficacy analysis

|  | | | Patients with IDH1 mutation | | Patients with IDH2 mutation | |
| --- | --- | --- | --- | --- | --- | --- |
|  |  |  | Number | Percent | Number | Percent |
| **CR rate** | | | | | | |
| Newly diagnosed AML | IDH inhibitor monotherapy group | | 33 | 45.83% | 39 | 54.17% |
|  | IDH inhibitor combination therapy group | | 155 | 48.29% | 166 | 51.71% |
| R/R AML | IDH inhibitor monotherapy group | | 125 | 22.20% | 438 | 77.80% |
| **ORR rate** | | | | | | |
| Newly diagnosed AML | | IDH inhibitor monotherapy group | 33 | 45.83% | 39 | 54.17% |
|  |  | IDH inhibitor combination therapy group | 155 | 49.36% | 159 | 50.64% |
| R/R AML | | IDH inhibitor monotherapy group | 125 | 22.20% | 438 | 77.80% |
| **2-year OS rate** | | | | | | |
| Newly diagnosed AML | | IDH inhibitor monotherapy group | 33 | 45.83% | 39 | 54.17% |
|  |  | IDH inhibitor combination therapy group | 132 | 45.36% | 159 | 54.64% |
| R/R AML | | IDH inhibitor monotherapy group | 125 | 22.20% | 438 | 77.80% |
| **Median OS** | | | | | | |
| R/R AML | | IDH inhibitor monotherapy group | 125 | 22.20% | 438 | 77.80% |
| **2-year EFS rate** | | | | | | |
| Newly diagnosed AML | | IDH inhibitor combination therapy group | 72 | 51.43% | 68 | 48.57% |
| **Median EFS** | | | | | | |
| R/R AML | | IDH inhibitor monotherapy group | NA | | 438 | 100.00% |

IDH: isocitrate dehydrogenase; CR: complete remission; ORR: overall response, including complete remission, complete remission with incomplete hematologic or platelet recovery, partial remission, and morphologic leukemia-free state; OS: overall survival; EFS: event-free survival; NA: efficacy analysis was not performed due to few or no included studies; AML: acute myeloid leukemia. R/R: Relapsed or refractory.
